# A granular look at solid electrolyte interfaces in lithium-ion batteries

**DOI:** 10.1038/s42004-021-00521-2

**Published:** 2021-05-25

**Authors:** Teresa S. Ortner

**Affiliations:** Communications Chemistry, https://www.nature.com/commschem

## Abstract

Lithium-ion batteries suffer from declining performance when the electrolyte decomposes. Now, low-dosage cryogenic transmission electron microscopy (cryo-TEM) visualizes how the common solid electrolyte interface component lithium carbonate decomposes and how additives stabilize the interface.

A solid electrolyte interface (SEI) forms upon initial charging of a liquid-electrolyte lithium-ion battery. SEI stability plays a prominent role for battery lifetime, but probing the intricate processes happening at the native SEI is a delicate endeavor. Now, Yonghong Deng, Ju Li, and Meng Gu from Southern University of Science and Technology, Shenzhen, China, and MIT, Cambridge, USA provide insights into native SEI compositions (10.1002/adma.202100404)^1^.

Common solid electrolyte interface components, such as lithium carbonate Li_2_CO_3_ and lithium sulfate Li_2_SO_4_, were long thought to be in direct contact with the metallic lithium electrode, acting as an electronic insulator. Thermodynamically, however, neither Li_2_CO_3_ nor Li_2_SO_4_ are stable in direct contact with a lithium anode in conventional lithium-ion batteries^2^. Recent technological progress in cryogenic transmission electron microscopy (cryo-TEM) now allows researchers to characterize sensitive chemical phases by eluding high-energy electron beams which damage the interface layer. Locations of randomly distributed inorganic and organic compounds can be resolved at the atomic scale^3^. “The biggest challenges are finding the right ultra-low dose electron imaging conditions and figuring out the cryo-transfer process that allows us to probe the native state of the SEI,” explains Meng Gu.

The team studied the interface evolution (Fig. 1) between lithium anodes and ethylene diethyl carbonate electrolytes, with and without 2% ethylene sulfate or 2% propane sulfonate additives. They found that formed Li_2_CO_3_ decomposes upon contact with the metallic lithium anode, as well as in the outer interface region, where it causes bubbles and formation of a porous SEI. Three amorphous phases were identified: an outermost organic polymeric phase, an oxide-sulfide phase in the middle, and a metallic lithium-rich phase in the interior, with increasing electronic conductivity closer to the metal electrode. Crystalline phases of Li_2_CO_3_, Li_2_SO_4_, and Li_2_O were dispersed within these amorphous matrices.

**Fig. 1:**
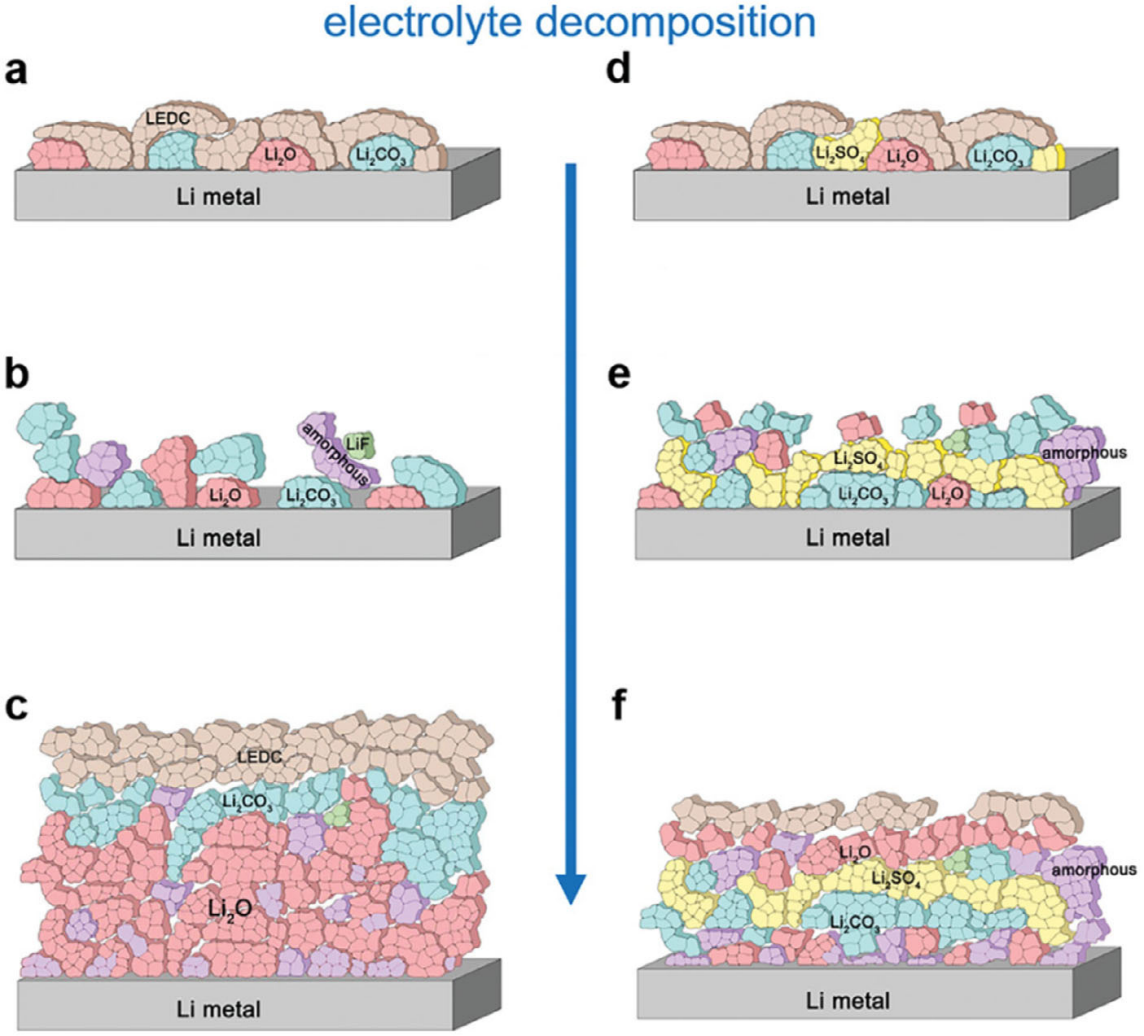
Schematic representation of SEI formation on a lithium metal electrode. Panels **a**–**c** show SEI evolution without additives in the ethylene diethyl carbonate electrolyte. Due to continuous decomposition of the formed lithium ethylene dicarbonate (LEDC) and lithium carbonate (Li_2_CO_3_), lithium oxide (Li_2_O) accumulates and covers the electrode. Panels **d**–**f** depict the effects of ethylene sulfate as an electrolyte additive. Lithium-sulfur species such as Li_2_SO_4_ form a layer (yellow) preventing further decomposition of Li_2_CO_3_. Reproduced from *Adv. Mater*. 2100404 (2021), copyright (2021) *Wiley‐VCH GmbH*.

Electrolytes with sulfur-containing additives show superior performance^4^ because Li_2_SO_4_ and Li_x_S encapsulate Li_2_CO_3_ and limit interface thickening, ultimately enhancing battery life. “We still need to find out so much more — such as the 3D structure of the SEI after lithium stripping, or the composition and functional groups in amorphous organic/polymeric matrices,” says Gu, “and cryo-TEM can play a significant role in clarifying all the potential functions of the SEI.”
